# Comparative transcriptomics of *Aspergillus fumigatus* strains upon exposure to human airway epithelial cells

**DOI:** 10.1099/mgen.0.000154

**Published:** 2018-01-18

**Authors:** Tonya N. Watkins, Hong Liu, Matthew Chung, Tracy H. Hazen, Julie C. Dunning Hotopp, Scott G. Filler, Vincent M. Bruno

**Affiliations:** ^1^​ Institute for Genome Sciences, University of Maryland School of Medicine, Baltimore, MD 21201, USA; ^2^​ Division of Infectious Diseases, Los Angeles Biomedical Research Institute at Harbor-UCLA Medical Center, Torrance, CA, USA; ^3^​ David Geffen School of Medicine at UCLA, Los Angeles, CA, USA

**Keywords:** *Aspergillus fumigatus*, RNA-seq, airway epithelial cells, Af293, CEA10

## Abstract

*Aspergillus fumigatus* is an opportunistic, ubiquitous, saprophytic mould that can cause severe allergic responses in atopic individuals as well as life-threatening infections in immunocompromised patients. A critical step in the establishment of infection is the invasion of airway epithelial cells by the inhaled fungi. Understanding how *A. fumigatus* senses and responds to airway cells is important to understand the pathogenesis of invasive pulmonary aspergillosis. Here, we analysed the transcriptomes of two commonly used clinical isolates, Af293 and CEA10, during infection of the A549 type II pneumocyte cell line *in vitro*. We focused our RNA-seq analysis on the core set of genes that are present in the genomes of the two strains. Our results suggest that: (a) *A. fumigatus* does not mount a conserved transcriptional response to airway epithelial cells in our *in vitro* model and (b) strain background and time spent in the tissue culture media have a greater impact on the transcriptome than the presence of host cells. Our analyses reveal both common and strain-specific transcriptional programmes that allow for the generation of hypotheses about gene function as it pertains to pathogenesis and the significant phenotypic heterogeneity that is observed among *A. fumigatus* isolates.

## Data Summary

All of the raw sequencing reads from this study have been submitted to the NCBI Sequence Read Archive (SRA) under BioProject; accession number: PRJNA399754 (url - https://www.ncbi.nlm.nih.gov/bioproject/PRJNA399754). The specific sample accession numbers are presented in Table S1 (available in the online version of this article).

**Impact Statement**

*Aspergillus fumigatus* is an opportunistic, ubiquitous, saprophytic mould that can cause life-threatening infections in immunocompromised patients. Understanding how *A. fumigatus* senses and responds to airway cells is important to understand the pathogenesis of invasive pulmonary aspergillosis. Here, we analysed the transcriptomes of two commonly used clinical isolates, Af293 and CEA10, during infection of the A549 type II pneumocyte cell line *in vitro*. Our analyses reveal both common and strain-specific transcriptional programmes that allow for the generation of hypotheses about gene function as it pertains to pathogenesis and the significant phenotypic heterogeneity that is observed among *A. fumigatus* isolates.

## Introduction

*Aspergillus fumigatus* is a ubiquitous environmental mould that releases numerous small conidia into the air. Inhalation of *A. fumigatus* conidia can cause a wide range of clinical manifestations depending on the immunological competency of the host [1, 2]. Hallmarks of invasive aspergillosis (IA) include angioinvasion that leads to tissue thrombosis and tissue infarction [3]. Although infections by *A. fumigatus* have been described in other sites of the body, the respiratory tract is the main route of entry and site of disease [4]. Invasive pulmonary aspergillosis (IPA) is a life-threatening condition that particularly occurs in patients with neutropenia. IPA is associated with a 25–35 % mortality rate despite current antifungal therapy [5, 6]. In a hospital survey, *A. fumigatus* represented 1 % of airborne mould conidia but accounted for nearly 50 % of patient isolates [7].

Infection is initiated when airborne conidia are inhaled and adhere to the epithelial cells that line the alveoli. The conidia then invade the host cells by a phenomenon known as induced endocytosis [8–13]. For *A. fumigatus*, this process is initiated by the interaction of the conidia cell-wall protein, CalA, with the integrin α_5_β_1_ expressed on the host cell plasma membrane [14]. Gliotoxin and β−1,3-glucan, via their ability to stimulate host phospholipase D activity, also contribute to the internalization of *A. fumigatus* conidia by A549 cells [15, 16]. Once endocytosed, conidia traffic to endosomes/lysosomes where germination is delayed. In some instances, these hyphae escape and because of their tropism for blood vessels, they are able to penetrate and cause damage to endothelial cells followed by extravascular invasion of deep organs [17].

A detailed understanding of the molecular mechanisms underlying *A. fumigatus* pathogenesis has been hampered by the extensive genetic and phenotypic heterogeneity among strains examined [18–20]. Specifically, there are reproducible differences in virulence among several *A. fumigatus* isolates as assessed by murine models of IPA [18, 20]. Furthermore, extensive variation in the immune response to different clinical isolates has also been observed [21]. Perhaps the most notable example in the literature is the difference between the two most commonly used ‘wild-type’ isolates, Af293 and CEA10 (previously A1163). CEA10 is more virulent than Af293 in a murine triamcinolone model of IPA [20] and there are also significant differences in the immune responses elicited by the two strains [21]. These studies underline the importance of using observations from multiple isolates to fully understand potential pathogenic mechanisms.

As lung epithelial cells are among the first host cells that *Aspergillus* conidia encounter during infection, a complete understanding of how these fungi respond to these cells is critical to understanding the pathogenesis of IPA. Previous studies have used microarrays to study the transcriptional response of *A. fumigatus* to airway epithelial cells [1], although the full catalogue of transcriptional changes is probably incomplete owing to the limited sensitivity and dynamic range of microarrays as well as the use of a single *A. fumigatus* strain. In this study, we used RNA-seq to examine the transcriptome of *A. fumigatus* exposed to the A549 type II pneumocyte cell line. To identify conserved and strain-specific transcriptomic programmes, we generated and compared transcriptomic data for strains Af293 and CEA10. Our results reveal both common and strain-specific transcriptional programmes that potentially shed light on the fungal host interaction and explain some of the phenotypic heterogeneity observed between the two strains.

## Methods

### *In vitro* infection

*Aspergillus fumigatus* strains Af293 and CEA10 were grown on Sabouraud agar (Difco) at 37 °C for 5–7 days prior to use. The conidia were harvested by rinsing the plate with PBS containing 0.1 % Tween 80 (Sigma-Aldrich) and enumerated using a haemacytometer. For use in the experiments, the A549 type II pneumocyte cell line [American Type Culture Collection (ATCC)] was grown in F-12 K medium (ATCC) containing 10 % FBS (Gemini Bio-Products) and 1 % streptomycin and penicillin (Irvine Scientific) in 5 % CO_2_ at 37 °C. The day before the experiments, approximately 7×10^5^ A549 cells in 2 ml of growth medium were cultured in each well of a six-well tissue culture plate. The next day, the A549 cells were gently rinsed twice with serum-free F-12 K medium. Next, 1×10^6^ conidia of each strain in 2 ml of F-12 K medium were added to individual wells of the six-well tissue culture plate. Control wells contained uninfected A549 cells and organisms incubated in F-12 K medium in the absence of A549 cells. After 6 and 16 h of incubation, RNA was extracted from the A549 cells and from *A. fumigatus* strains using the RiboPure Kit (Ambion, Life Technology) and purified using the RNA Clean and Concentrator Kit (Zymo Research).

### RNA-seq data generation

All RNA-seq libraries (non-strand-specific, paired end) were prepared with the TruSeq RNA Sample Prep kit (Illumina). The total RNA samples were subject to poly(A) enrichment as part of the TruSeq protocol. In total, 100 nt of sequence was determined from both ends of each cDNA fragment using the HiSeq platform (Illumina) as per the manufacturer’s protocol.

### Gene expression analysis

In order to identify 1 : 1 homologues shared between the Af293 and CEA10 strains, genes were clustered into Jaccard orthlogous clusters (JOCs) [22]. JOCs were associated with InterPro descriptions and gene ontology (GO) terms using InterProScan v5.22.61.0 when all genes within the cluster shared the functional description [23].

Sequencing reads were annotated and aligned to the *A. fumigatus* reference genomes (Af293 and CEA10) using TopHat2 [24]. The percentage of total reads that mapped to *A. fumigatus* was appreciably lower when the human cells were present (Table S1). This is expected as the sequencing library preparation involves a poly(A) enrichment step that is unable to separate fungal-derived transcripts from human-derived transcripts. The alignment files from TopHat2 were used to generate read counts for each gene. For the clustering analysis, EdgeR v.3.14 [25] was used to fit read counts per coding sequence to a quasi-likelihood fit and a false discovery rate (FDR) <0.01 threshold was used to identify 7888 differentially expressed genes from a total of 9041 JOCs. Raw read counts were converted to a transformed normalized count value (transcripts per kilobase million, TPM) to normalize for gene length and the library size for each sample. A k-means clustering approach as implemented in the k-means function of R v3.3.1 was used on the TPM value for each gene in each sample for all differentially expressed genes to identify clusters of genes with the same transcriptional response. The appropriate number of clusters to use was determined by plotting the WSS (within-cluster sum of squares) as a function of the number of clusters and chosing the number of clusters beyond which the intra-cluster variation no longer decreased. Heat maps of the different k-means clusters were drawn using the log_2_(TPM) value for each gene using the gplots v3.0.1 R package. Enriched InterPro descriptions and GO terms within each cluster were defined using a Fisher’s exact test *P*-value <0.01 as implemented in the fisher.test function in R v3.3.1. A principal component analysis (PCA) was done with the prcomp function in the R stats package using the TPM values for each of the genes in all samples. Each individual sample was labelled using different colours while time points were differentiated using shapes. Genes contributing to the first and second principal components were extracted using the R package factoextra v1.0.5 and enriched functional terms were determined as described above.

Differential expression analysis between specific experimental groups was performed using the EdgeR package from Bioconductor [25]. For these specific comparisons, a gene was considered differentially expressed if the FDR for differential expression was less than 0.01 and the absolute log_2_ fold-change (LFC) was greater than or equal to one. The circular displays of the significant LFC values were generated using Circos 0.69–5 [26].

GO term and Metabolic Pathway enrichment analyses were performed using the tools available at FungiDB (http://fungidb.org/fungidb/) using default settings [27]. VENNY2.1 was used to generate the Venn diagrams (http://bioinfogp.cnb.csic.es/tools/venny/)

## Results and discussion

### RNA-seq of i*n vitro A. fumigatus* infections

To understand the molecular nature of the interaction between fungal pathogen and host cells in the context of aspergillosis, we performed RNA-seq analysis on poly(A)-enriched RNA isolated from monolayers of airway epithelial cells (A549 cells) infected for 6 or 16 h with two well-characterized clinical isolates of *A. fumigatus*, strains Af293 and CEA10.

After being added to human cell culture, the resting conidia first swell and then, after approximately 4 h, begin to germinate. Following germination, the hyphae elongate rapidly and begin to branch such that the host cell monolayer is covered by a hyphal mat by 16 h after addition of the conidia (Fig. S1). This process occurs independently of the presence of host cells, so it is important to differentiate between transcriptional changes in the fungi that are specifically induced by interaction with the host cells from those that are part of a transcription programme that accompanies swelling, germination and mycelial growth. To accomplish this, RNA-seq was performed on time-matched controls in which each isolate was grown in tissue culture medium without host cells. Each of the eight experimental conditions (Table 1) was carried out in triplicate. The RNA preparations contained a mixture of mRNAs expressed by *A. fumigatus* as well as by the host cells. All sequencing reads were aligned to the reference genome (Af293 or CEA10). Analysis of the host transcriptome will be described in a separate publication. From each of the 24 sequencing libraries we obtained an average of 53±29.3 million reads that mapped to the appropriate fungal reference genome (Table S1).

**Table 1.** Experimental groups

Group	Host cells	*A. fumigatus* strain	Incubation time (h)
1	–	Af293	6
2	A549	Af293	6
3	–	Af293	16
4	A549	Af293	16
5	–	CEA10	6
6	A549	CEA10	6
7	–	CEA10	16
8	A549	CEA10	16

### Common and strain-specific responses to the *in vitro* growth conditions

**Fig. 1.**
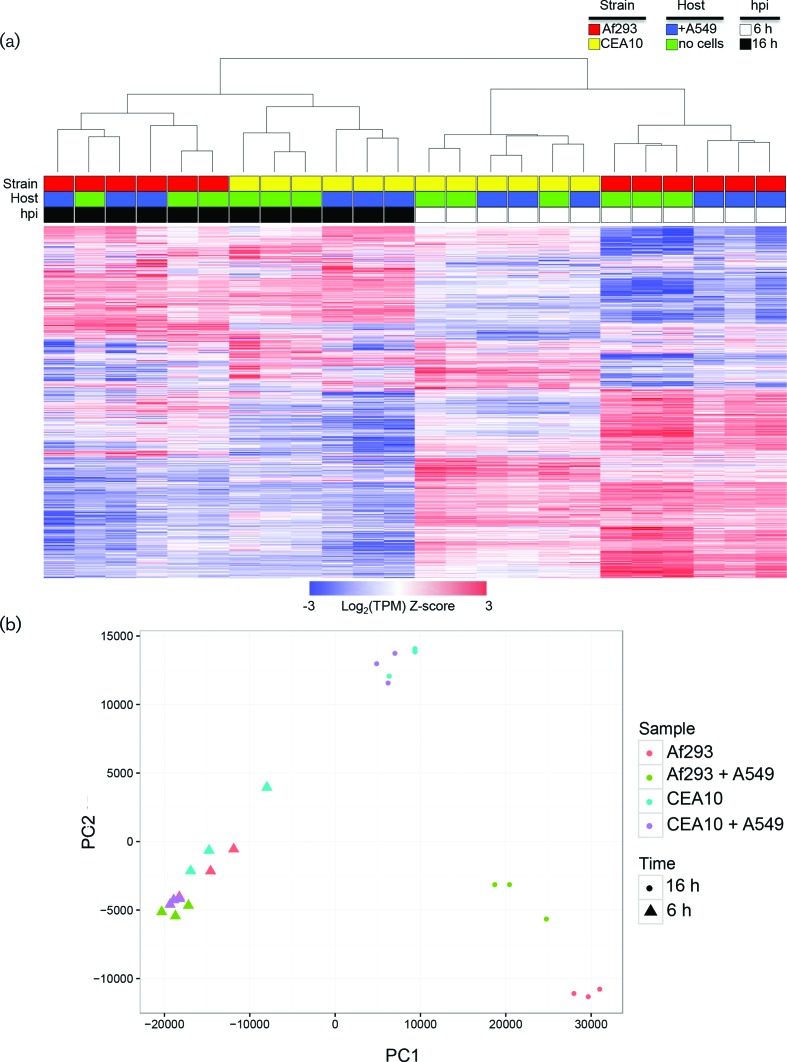
Global analysis of differential gene expression across all samples. (a) Hierarchical cluster analysis, based on log_2_-transformed TPM, of all 7888 differentially expressed *A. fumigatus* core genes after growth in tissue culture medium with or without host cells. Each column represents an individual sample (*n*=24) as designated by the colour code above the dendrogram. Each row represents a different gene. Red indicates high gene expression. Blue indicates low gene expression. (b) PCA of TPM values from all differentially expressed genes in all 24 samples.

While the genomes of these two *A. fumigatus* strains are highly similar and syntenic, they are not identical; each strain has many genes with no detectable orthologue in the other strain [28, 29]. We took a conservative approach to comparing the transcriptomes by focusing our analyses on the core set of genes that contain clear 1 : 1 homologues in both genomes. This allowed us to avoid strain-specific responses that stem from large-scale differences in the genomes. Using Jaccard cluster analysis, we defined a set of 9041 genes that we refer to as the ‘core’ genome (see Materials and Methods) (Table S2). In total, 7888 of these core genes were differentially expressed (FDR<0.01, regardless of fold-change size) in at least one experimental group compared to any of the other seven groups. For this set of 7888 genes, we performed hierarchical clustering of all 24 samples using the log_2_-transformed TPM values. Clustering analysis demonstrated that the samples grouped primarily by time spent in the tissue culture media, with additional grouping of samples based on strain (Fig. 1a). PCA confirmed the groupings determined by hierarchical clustering (Fig. 1b).

The top genes, contributing to 95 % of the principal components, were subjected to a functional term enrichment analysis. The 163 genes with the highest contribution to the first principal component are overrepresented in functional terms relating to the ribosome and translation (GO:0005840, GO:0006412) (Table S3). Similarly, the 171 genes with the highest contribution to the second principal component were enriched for ribosomal and translational functional terms, albeit less than the first principal component, while also being enriched for proteins involved in the electron transport chain (GO:0004129, GO:0015986) (Table S4). These results indicate that the separation of the samples on the first and second principal components is due primarily to transcriptional differences in genes involved in translation and metabolism. Based on the experimental timing described above, these changes are likely to reflect a difference in fungal growth dynamics rather than a response to lung epithelial cells.

**Fig. 2.**
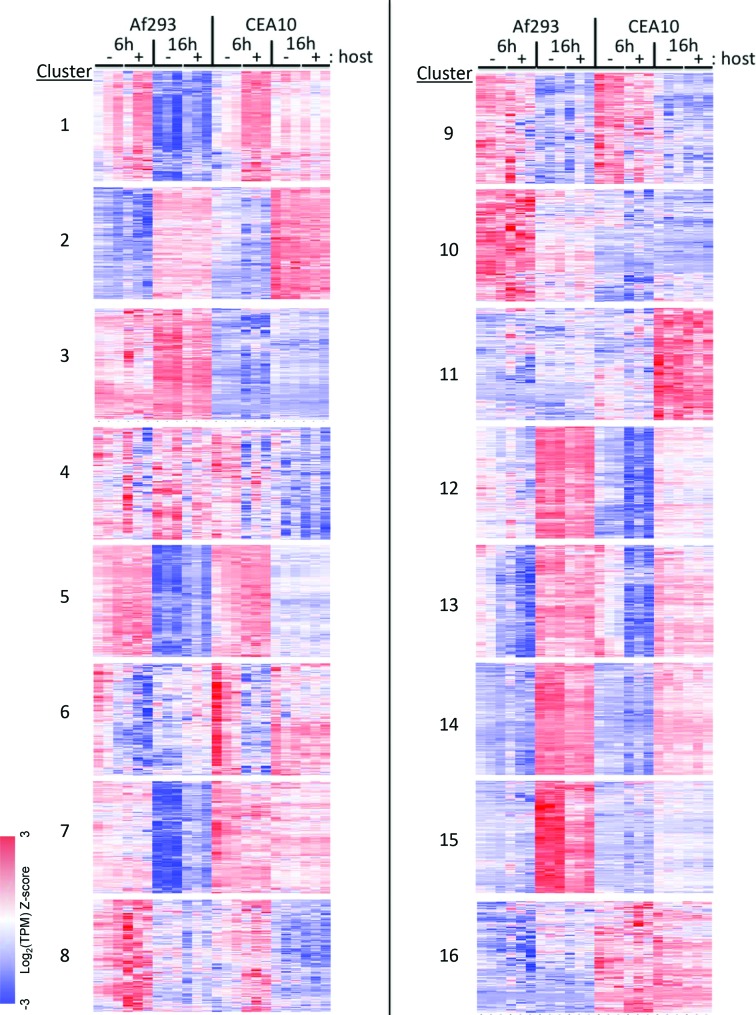
Heatmaps of clusters of differentially expressed genes. Groups of co-expressed genes revealed by k-means cluster analysis. Each column represents an individual sample (*n*=24). Values represent log_2_-transformed TPM values. Red indicates high gene expression. Blue indicates low gene expression. Clusters 12, 14 and 5 represent common responses to time in the culture model independent of the presence of host cells. Clusters 15, 3 and 16 represent groups of genes with strain-specific patterns of gene expression.

To identify specific gene expression patterns and groups of co-regulated core genes, we performed k-means clustering on the list of 7888 differentially expressed genes (see Materials and Methods). We then performed GO and metabolic pathway enrichment analysis on the clusters of differentially expressed genes to gain insight into the biological processes that were regulated over the course of the experiment. Heatmaps of all clusters and results of the enrichment analyses are shown in Fig. S2 and Tables S5–S36, respectively. The genes clustered into 16 major groups, revealing both common and strain-specific responses to time spent in the tissue culture medium (Fig. 2). The gene membership for each of the 16 clusters as well as expression data for each gene in each sample can be found in Table S37. Three groups (clusters 2, 12 and 14) consisted of genes that are expressed at higher levels in the 16 h samples compared to the 6 h samples in both strains, independent of the presence of host cells (Fig. 2). Cluster 12 showed enrichment for genes involved in glucan and glycogen biosynthesis (Table S27) while cluster 14 was enriched for genes predicted to play a role in transmembrane transport and cell-wall organization (Table S31). Clusters 12 and 14 were both enriched for genes involved in retinol metabolism (Tables S28 and S32). Cluster 5 contained genes whose expression was significantly lower at 16 h as compared to 6 h, independent of the presence of host cells (Fig. 2). This cluster was enriched for genes that function in nitrogen compound metabolism and translation (Tables S13 and S14). In each of the clusters, the gene expression at a given time-point was equivalent in the presence or absence of host cells, indicating that the genes represent a common response to extended time in the tissue culture medium and are most likely part of a transcriptional programme that accompanies, or drives, the transition from spore swelling to extended mycelial growth.

We also identified four groups (clusters 1, 7, 11 and 15) that reveal host cell-independent, strain-specific responses to time in the tissue culture media (Fig. 2). Notably, cluster 15 contains genes whose expression increases over time only in strain Af293 and is highly enriched for genes involved in secondary metabolite biosynthetic process (*P*=8.08e-17). Included in this cluster are 11 genes involved in fumagillin biosynthesis and eight genes involved in gliotoxin biosynthesis (Table S33). These results suggest that Af293 might produce more secondary metabolites than CEA10 during mycelial growth in our experimental conditions, but further experiments are required to confirm this.

Finally, we obtained two groups that reveal constitutive strain-specific differences in gene expression. Cluster 3 contains genes that are more highly expressed in strain Af293 while cluster 16 contains genes that are more highly expressed in strain CEA10 (Fig. 2). The expression of genes in both these clusters is independent of exposure to host cells as well as to time spent in the media. Cluster 3 is enriched for genes that are involved in alcohol catabolism and the response to hydrogen peroxide (Table S9). These clusters hold the potential to provide insight into phenotypic differences between Af293 and CEA10 by providing clues about strain-specific gene expression. Notably, our clustering analysis did not reveal any clusters of *A. fumigatus* genes that are differentially expressed in response to exposure to A549 cells. Taken together, the global patterns of gene expression that we observe suggest that, in our *in vitro* model of *A. fumigatus* infection of airway epithelial cells, strain background and time spent in the tissue culture media have a greater impact on the global transcriptional output than the presence or absence of host cells.

### Transcriptional responses to host cell exposure

**Fig. 3.**
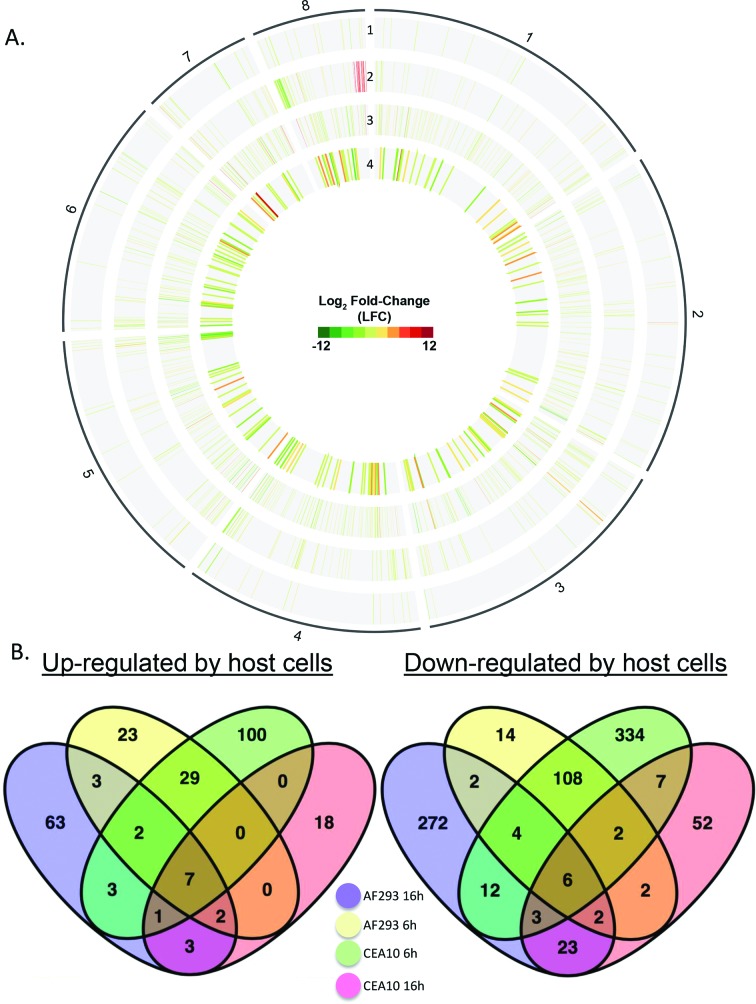
Differential expression of genes in response to host cells. (a) Circos plot comparing the host-cell-induced differential expression (FDR<0.01; LFC≥|1.0|) of the two *A. fumigatus* strains at each time-point of infection. The four numbered tracks represent individual comparisons of infection sample groups with time-matched negative controls: Track 1, CEA10 at 16 h post-infection (h p.i.); track 2; Af293 at 16 h p.i.; track 3, CEA10 at 6 h p.i.; track 4, Af293 at 6 h p.i. Red represents up-regulation in response to incubation with host cells. Green represents down-regulation under the same conditions. Genes that did not exhibit significant differential expression are absent from this plot. Chromosomal location is indicated by the outer black line and the adjacent numbers (1–8). (b) Venn diagrams representing the overlap in gene regulation between the two strains.

Given our inability to identify groups of fungal genes that responded to the epithelial cells using k-means clustering, we focused on specific comparisons of the groups of co-cultured samples with the appropriate time-matched negative control groups. For this analysis, we defined differentially expressed genes as those with a minimum of two-fold change in gene expression (FDR<0.01). Each of the *A. fumigatus* strains mounted a modest transcriptional response to A549 cells. Strain Af293 displayed 207 and 408 differentially expressed genes at 6 and 16 h post-infection, respectively. Under these same experimental conditions, strain CEA10 displayed 619 and 128 differentially expressed genes, respectively. Strain CEA10 exhibited a stronger response to 6 h of exposure to A549 cells while strain Af293 mounted a stronger response at the 16 h time-point (Fig. S2, Table S38). Relatively few core genes were regulated in the same direction in both strains (Fig. 3). In fact, 70 % of the differentially expressed genes were strain-specific when only matching time-points were considered. We also took into account the possibility that the dynamics of gene induction or mRNA stability of a given gene may vary between the two strains. To this end, we found that only 8.6 % of the gene regulations occurred in the same direction in both strains but at different time-points (Fig. 3b). Collectively, these results indicate that we were unable to detect a highly conserved transcriptional response to airway epithelial cells in these two *A. fumigatus* strains. Furthermore, the genes that govern adherence and invasion of host cells appear to be constitutively expressed during growth in tissue culture media alone, independent of exposure to host cells.

**Fig. 4.**
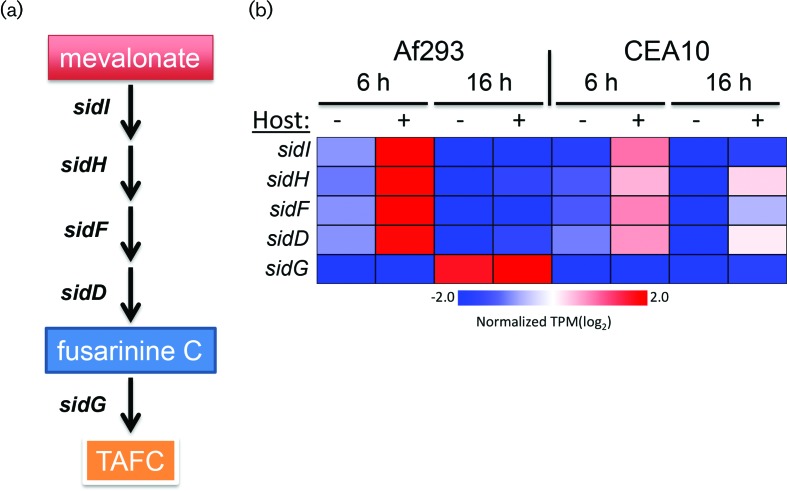
Fusarinine C biosynthetic genes are induced during exposure to A549 cells. (a) Simplified schematic of FSC and TAFC biosynthesis by the enzymatic activities of the *sid* genes (adapted from [32]). (b) Expression of FSC biosynthetic genes in each of the eight experimental groups. Values represent the average of the log_2_-transformed normalized TPM values for each group. Red indicates high gene expression. Blue indicates low gene expression. The expression of sidG, required for conversion of FSC to TAFC, was not induced by exposure to host cells.

Despite our inability to detect a conserved response to airway epithelial cells, we were able to detect some predicted functional enrichment among the genes that were regulated in the same manner in both of the strains. Since the lists had relatively few genes, we analysed the set of 47 genes that were up-regulated in both strains during at least one of the time-points, allowing us to include genes whose temporal dynamics might vary between the strains. This set of genes was enriched for ‘*N*’,*N*’,*N*’-triacetylfusarinine C biosynthetic process’ (*P*=6.38e-6). Triacetylfusarinine C (TAFC) and fusarinine C (FSC) are hydroxamate-type siderophores used by *Aspergillus* to acquire iron [30]. FSC, the immediate precursor to TAFC, is generated from melavonate by four steps that include the enzymatic activities of sidI, sidH, sidF and sidD [31, 32]. FSC is crucial for virulence because deletion of any of the FSC biosynthetic genes attenuates virulence [31, 32]. We observed the up-regulation of sidI, sidH, sidF and sidD in response to A549 cells in both strains (Fig. 4). sidC, sidD, sidF and sidG are known to be induced by iron-starvation [31, 32]. Our inability to detect up-regulation of sidC and sidG in our experiments suggests that the fungi may not be experiencing iron-starvation but rather responding to a host-cell-derived signal which may serve as a warning for pending iron-starvation. Additional experiments are required to determine the molecular nature of the signal that induces the gene expression changes.

The true utility of this dataset stems from its ability to help generate hypotheses about which genes govern virulence. This is particularly important given the dearth of drugs available to treat IPA and the relatively small list of genes (~40–50) that are known to be required for full virulence in murine models. Strain-specific changes in gene expression provide testable hypotheses about the molecular mechanisms underlying the strain-to-strain variability in virulence, while conserved gene expression changes have the potential to reveal more fundamental virulence mechanisms and provide drug targets that will be effective against a variety of *A. fumigatus* strains. We observed 89 genes whose expression was only induced upon exposure to host cells in Af293 and 118 genes whose expression was specifically induced in CEA10. The majority of the genes with strain-specific changes in gene expression are uncharacterized and have untested roles in virulence, so future follow-up studies are necessary to determine if these differences contribute to the documented variation in virulence and immunogenicity. Among these genes, 53 (26 for Af293 and 27 for CEA10) are predicted to have secretion signals (Tables S39 and S40). These genes are of particular interest as their potential extracellular localization makes them more likely to have a direct role in the fungal–host interaction. Complete lists of strain-specific gene inductions are presented in Tables S39 and S40.

If we consider the list of 47 genes that are up-regulated by exposure to host cells in both strains, mutants disrupted for 10 of those genes have been tested in an animal model of aspergillosis. Remarkably, seven out of those 10 mutants have attenuated virulence (Table S41). Among the other 37 genes from this group, 13 are predicted to have secretion signals and two have predicted cell-wall localization (Table S41). These results provide a manageable subset of genes to test for a role in virulence and suggest the high probability of positive identification. For both the strain-specific and the conserved lists, further functional studies in multiple genetic backgrounds are required to determine if these genes contribute to pathogenesis and/or the variability in virulence.

To our knowledge, only one study has examined the response of *A. fumigatus* to lung epithelial cells [1]. The list of genes that we found to be differentially expressed does not overlap significantly with this study. For example, of the 69 genes that are reported to be differentially expressed by Oosthuizen *et al.* [1], only seven changed in the same direction in at least one time-point in our study. There are several possible explanations for the poor concordance. Oosthuizen *et al.* used a microarray platform to examine the transcriptome response of polarized 16HBE14o- bronchial epithelial cells to a different *A. fumigatus* strain (a GFP-expressing derivative of ATCC 13073) used in our study. So, the most likely explanation for the discordance between the two studies is the combination of different host cells, different *A. fumigatus* strains and the well-established differences between microarrays and RNA-seq.

## Data bibliography

1. University of Maryland Institute for Genome Sciences. Sequence Read Archive - PRJNA399754 (2017).

## Supplementary Data

643 Supplementary File 1

## Supplementary Data

7067 Supplementary File 2
