# Supplementary material for: Comparative transcriptomics of Aspergillus fumigatus strains upon exposure to human airway epithelial cells
Source: Microb Genom. 2018 Jan 18;4(2):e000154. doi: 10.1099/mgen.0.000154 (PMC5857381; doi:10.1099/mgen.0.000154)

Figure S1. Interaction of *A. fumigatus* with A549 cells. Microscopic images of A549 cells infected with each strain of *A. fumigatus* for 6 for 16 h *in vitro*.

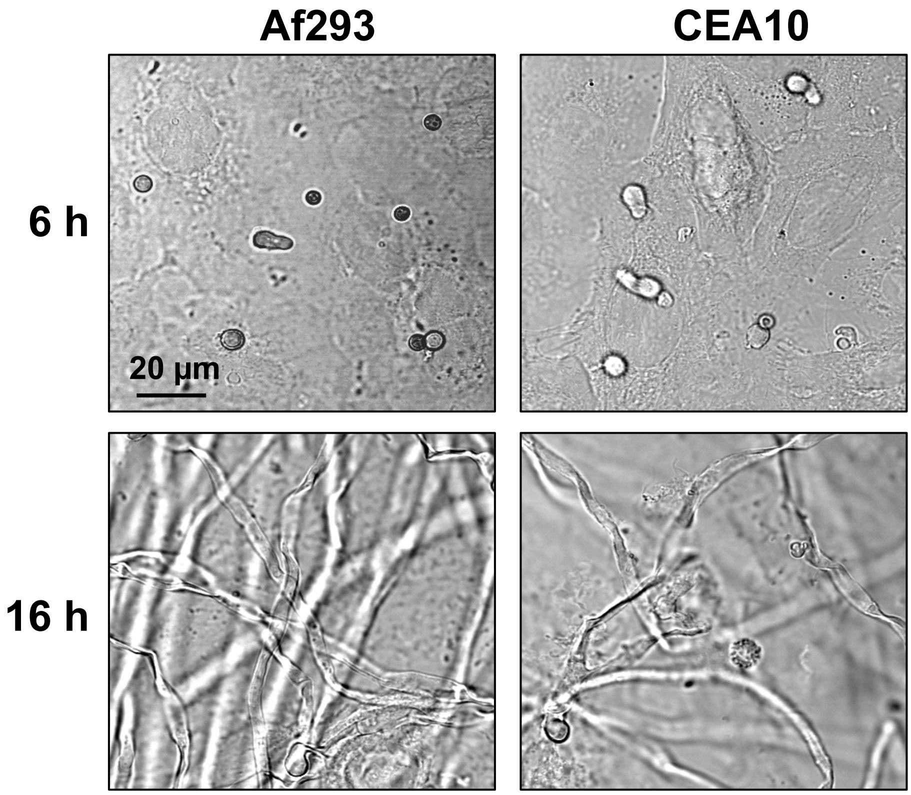

Figure S2. Differential expression of *A. fumigatus* genes. The number of genes that were induced (yellow) or repressed (blue) by exposure to A549 cells at 6 or 16 h after addition to co-culture.

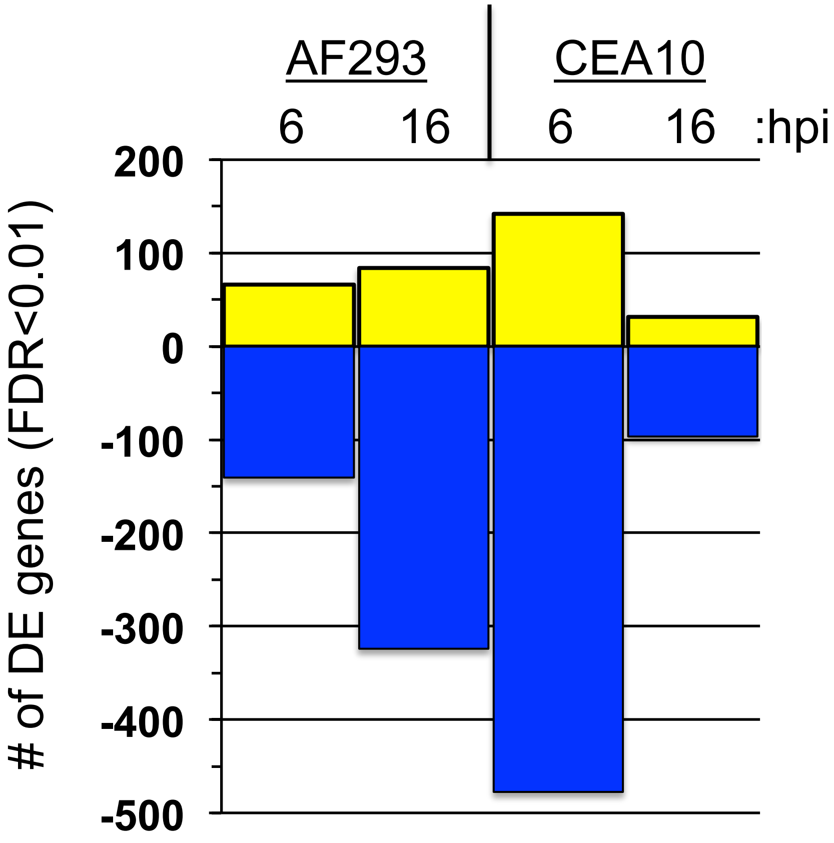

Supplement: Supplementary File 1 [file mgen-4-154-s001.pdf]
